# Work-related stress and associated factors among employees of Hawassa industrial park, southern Ethiopia: an institutional based cross-sectional study

**DOI:** 10.1186/s12888-022-04032-9

**Published:** 2022-06-07

**Authors:** Yohanes Sime, Hailemariam Hailesilassie, Arefayne Alenko

**Affiliations:** 1 Department of Psychiatry, College of Medicine and Health Science, Dilla University, Dilla, Ethiopia; 2 Psychiatry Department, Faculty of Medical Sciences, Jimma University, Jimma, Ethiopia

**Keywords:** Work-related stress, Employee, Industrial park, Ethiopia

## Abstract

**Background:**

Work-related stress (WRS) is becoming an alarmingly growing public health concern worldwide. Due to globalization and changes in working conditions, people in low-income countries face growing work-relates stress. However, despite high prevalence globally, work-related stress among industrial park workers is not well studied in Ethiopia.Thus; the aim of this study was to assess work-related stress and associated factors among employees of Hawassa industrial park, southern Ethiopia.

**Methods:**

An institutional-based cross-sectional study was employed among 419 employees of Hawassa industrial park using an interviewer-administered structured questionnaire. Study participants were selected using simple random sampling technique. Data was collected by face-to-face interview. A workplace stress scale (WPSS) was used to assess work-related stress. The collected data were coded and entered into EPI data 4.6 and exported to SPSS version 26 for analysis. Multivariable logistic regression analysis was conducted to identify associated factors. The statistical significance was considered at *P*-value < 0.05.

**Result:**

The Overall prevalence of work-related stress was 47.5, 95% CI (43.2, 52.1). Variables such as temporary employment [AOR = 0.41, 95% CI (0.26–0.64)], poor working condition [AOR = 2.12, 95% CI (1.32–3.43)], work experience less than two and half years [AOR = 3.11, 95% CI (1.95–4.96)], poor learning opportunity [AOR = 1.82, 95% CI (1.10–2.30)], poor organizational support [AOR = 1.70, 95% CI (1.10–2.62)], current use of khat [AOR = 2.52, 95% CI (1.28–4.99)] and current use of alcohol [AOR = 2.27, 95% CI (1.44–3.58)] were significantly associated with work-related stress.

**Conclusion and recommendation:**

The study found high prevalence of work-related stress among employees of Hawassa industrial park. Temporary employment, poor working conditions, work experience < 2^1^/_2_ years, poor learning opportunities, poor organizational support, current khat use, and current use of alcohol were significantly associated with work-related stress. Our study finding is recommending enhancing stress management skills and primary prevention on identified risk factors to industry employees.

## Introduction

Stress is a sensation of mental pressure and tension in psychological sciences. Low-stress levels may be desirable, useful, and even healthy to improve bio-psychosocial health and improve performance in its positive form. However, high stress can lead to biological, psychological, and social issues and even serious damage to people [1]. A number of people who have stress caused or made themselves worse through work increases at an alarming rate and in developing countries, it becomes an issue of public health concern [2].

Work-related stress (WRS) is a harmful physical and/or emotional response when the needs of a job do not correspond with the employee’s abilities, resources, or needs [3]. Work-related stress occurs if the requirements of the job differ from the individual worker’s resources and abilities to meet these requirements. Next to musculoskeletal disorders, WRS is the second most reported work-related health problem [2].

WRS causes various health concerns and impropriety. The most common health concerns include back pain, muscle aches, headache, stomach ache, bloated stomach, constipation, high blood pressure, heart problems, depression, anxiety, fatigue, annoyance, asthma [4]. In addition, the quality and productivity of work decrease with these negative developments, and disease and absence increase [5].

Globally, work-related stress is a major challenge to workers and also organizations. It affects the mental and physical health of an individual and the effectiveness of an organization [6]. In recent decades, globalization and technological progress have changed the world of work, introducing new forms of work organization, working relations, and employment patterns and contributing to the enhancement of WRS [7]. Industrial park workers are an important health prevention population, including the prevention of mental health problems especially stress which is related to their work [8].

The magnitude of recorded work-related stress has increased over the years, and the losses for organizations and businesses have escalated afterward too. It was found that up to 40% of the cost of losing the gross domestic product per annum from 0.5 to 3.5%, could be attributed to WRS [9]. The prevalence of work-related stress among employees of manufacturing sectors is high even though it varies across countries. It has been found to be 27.5% in Thailand [10], 23.9 in China [11], 25% in India [12], 21.3% in Iran [13], 28% in the Democratic Republic of Congo [14], and 45.2% in Ethiopia among employees in textile factory [15].

Prior findings from the research have shown that the risk, severity, and impact of stress associated with the work have differed, depending on cultural orientation, work nature, and working environment. Some of the factors that contributed to WRS among employees were the shift work, the use of a psychoactive substance, social support, over 50 h of work per week, long daily working hours, high work demands, time pressure, and too many administrative tasks socio- professional factors, and demographic factors such as age [13, 16–18].

Work-related stress management results in work productivity, improved labor efficiency, reduced absenteeism, sound co-operation and friendly relations with all colleagues, and good achievement of mission and vision. Some mechanisms for stress management to be used within the different organizations include a redesign of work, training in stress management; environmental design; developing stress management training, and developing organizational systems for better work and management [19]. However, in our country and also Hawassa industrial park those activities are not well known and applied.

In developing countries, the magnitude of work-related stress and its determinants are one of the major public health problems [20]. In Ethiopia, even though the investment of industrial zones has become increasing in the past 10 years, little is known about the magnitude and the determinants of the problem on the most at-risk manufacturing industry workers. Therefore, we conducted this study to assess work-related stress and its associated factors among employees of Hawassa industrial park, as baseline study.

## Method and materials

### Study design

An institutional-based cross-sectional study design was employed at Hawassa industrial park.

### Study area and period

The study was conducted at Hawassa industrial park, Hawassa city, Southern Ethiopia. The Hawassa Industrial Park, which opened in July 2016, has been described as the Ethiopian government’s “flagship” industrial park. It is found in Hawassa city, located 275 km from Addis Ababa, and the capital of Ethiopia. The city has a latitude and longitude of 7°3′N 38°28′E and an elevation of 1708 m (5,604 ft) above sea level. The company encompasses an area of 1.3 million square meters, of which 300,000-m square is a factory shed build-up area. Currently, 22 leading global apparel and textile companies from America, China, India, Sri Lanka as well as different local manufacturers are operating within the park [21]. Currently, the company has overall 13,700 workers across 52 shades and around 80% of them are female. The study was conducted from August 1 to 30, 2021.

### Source population

All employees work in Hawassa industrial park.

### Study population

Employees who were available in their workplace during the study period.

### Eligibility criteria

#### Inclusion criteria

The study included employees who were currently working in the industry park.

#### Exclusion criteria

Those employees who were acutely ill and couldn’t communicate during the data collection period were excluded from the study.

### Sampling procedure and sampling technique

#### Sample size estimation

The sample size required for the study was calculated using a single population proportion formula by considering an estimated prevalence of work-related stress to be 45.2% [15], from the study conducted in Bahir Dar Textile Factory north-west Ethiopia, a 5% margin of error, a 95% confidence interval, and 10% non-response rate.

$$\mathrm{n}=\frac{{\mathrm{Z}}^2\mathrm{P}\left(1-\mathrm{P}\right)}{{\mathrm{d}}^2}=\frac{1.96^20.452\left(1-0.452\right)}{0.05^2}$$

Where n is the sample size, P is expected prevalence (proportion) of work-related stress, d = margin of error and Z = standard score corresponds to 1.96.

Therefore the total sample size was determined by using the above formula

$$\mathrm{P}=45.2\%$$

$$\mathrm{Z}=1.96\ \mathrm{at}\ 95\%\mathrm{CI}$$

$$\mathrm{d}=5\%(0.05)$$

$$\frac{\mathrm{n}={1.96}^20.452\left(1-0.452\right)=380.62=381}{0.05^2}$$

By considering 10% non-response rate the final sample size for the study was *n* = 419.

#### Sampling procedure

First the total list of employees in the Hawassa industrial park was taken from the human resource office. Then a simple random sampling technique was employed through the computer generation random method by using Microsoft excel to select all 419 samples from the total list of employees. Finally the data was collected from the selected samples at their corresponding working place.

### Study variable

#### Dependent variable

Work - related stress (Yes/No).

#### Independent variable

##### Socio demographic factors

Age, Gender, Religion, Marital status, Educational status, Types of employment, Income, Family size, Types of work, Position at work.

##### Organizational factors

Working conditions, Job security, Experience in the current organization, Working hours, Organizational support, Employee recognition, Overtime work.

##### Job content factors

Time pressure, Job control, Opportunity to learn, Interactions with machines, Workplace violence, Physical environment.

##### Substance use

Alcohol use, Khat use, Tobacco use, Cannabis use, > 1 substance use.

##### Health related factors

Chronic medical illness and acute illness.

**Operational definition:**

- **Work related stress**: A sum score below 60 of workplace stress scale was classified as having a work-related stress among participants [22].
- **Working condition**: Poor working conditions was considered with the summed scores of participants’ on questions to assess working condition are less than 10 [16, 23].
- **Organizational support:** Poor organizational support considered with the summed scores of participants’ on questions to assess organizational support was less than 7 [23].
- **Temporary employee:** employees those who had no permanent contract or recognition letter as permanent employee from the organization.
- **Time pressure:** High time pressure was defined as the summed scores of participants’ on questions to assess time pressure more than 10 [16].

### Data collection tool and procedure

A 20-point standard questionnaire (WPSS) was used to measure work-related stress. The American Institute of Stress validated this tool, which is now used in a variety of occupations. It is a standard questionnaire with a 5-point Likert scale. The scores ranged from 1 (never) to 5 (very often). The inverse scores ranged between 5 (never) and 1 (very often). For all WPSS questions, the findings were summarized, and less than 60 scores were graded as work-related stress [24]. The internal consistency, cronbach’s alpha coefficient of WPSS in the current study was 0.83.

The Job Content Questionnaire (JCQ) [25], and the National Institute for Occupational Safety and Health (NIOSH) generic questionnaires [26], inquired about organizational factors (working conditions, overtime work, experience in the organization, working hours, organizational support, employee recognition, and job security), and job content factors (time pressure, job demand, job control, resources, opportunity to learn, interactions of people with machines, illness, and physical environment). A poor working condition is the summed scores of less than 10. Poor organizational support is the summed scores of less than 7. High time pressure is the summed scores of more than 10. The Poor physical environment is the summed score of below 9. These instruments were used in a previous study conducted among bahirdar textile factory and dukem shoe factory employees in Ethiopia and it was valid and reliable [15, 16].

Screening Test for Alcohol, Smoking, and Substance (ASSIST Version 2.0) is made up of eight items that measure lifetime(Question 1 rated “yes” = 1/“no” = 0; interview stops if “no”) and recent (past 3 months; question 2: interview continues for each substance used in the past 3 months only) of substance. After reviewing different literatures four questions adopted from the tool to assess thr use of tobacco, alcohol, Khat and cannabis [27].

The data were collected through an interviewer-administered structured questionnaire, through a face-to-face interview by Amharic and English language. Four data collectors (Bsc nurse) were employed for one-month data collection periods and supervised by two supervisors (BSc psychiatry professional). The training was given for 1 day regarding the administration protocol of the data collection procedures for the data collectors by the main investigator.

### Data processing, analysis and presentation

Before entering the data into the computer, it was checked for completeness and cleaned. The data was then coded, cleaned, and edited before being entered into EpiData version 4.6 and exported to SPSS version 26 for analysis. Frequency, tables, texts, and summary measures were used to present descriptive statistics. Using binary logistic regression, bivariate and multivariable analysis was performed to determine the relationship between each independent variable and the outcome variable. To control for all possible confounders, all variables with *P* value less than 0.25 in the bivariate analysis were included in the final model of multivariable analysis. At a *P*-value greater than 0.05, the Hosmer-Lemeshow statistic was used to assess the goodness of fit. The statistical associations were calculated using an odds ratio with a 95% confidence interval. Using multivariate analysis in binary logistic regression, the adjusted odds ratio and 95% CI were calculated to identify the associated factors with work-related stress. A P-value of < 0.05 was considered statistically significant in this study.

## Result

### Socio demographic characteristics of study participants

From the total of 419 employees, 413 participated in the study giving a response rate of 98.6%. Most of the participants 295(71.4%) were female. The mean age of the participants was 26.7 (SD = 5.707) years. Of the study participants 189 (45.8%) were married and more than half 212 (51.3%) of the study participants were Protestant Christian religion followers. The majority 252(61.0%) of participants had a current educational status of above secondary school and about half 212 (51.3%) of the study participants were a temporary employees. More than three fourth 322 (78%) of the study participants were onsite workers and the majority 229(55.4%) of study participants had average monthly income (greater than 2629 ETB) (Table 1).

**Table 1:** Socio-demographic characteristics of employees in Hawassa industrial park in Hawassa city, South Ethiopia, September, 2021 (*N* = 413)

Variables	Categories	Frequency(n)	Percentage (%)
Sex	Male	118	28.6
	Female	295	71.4
Age	18–24	154	37.3
	25–34	216	52.3
	35–44	43	10.4
Marital status	Single	196	47.4
	Married	189	45.8
	Widowed	14	3.4
	Divorced	14	3.4
Current educational status	Illiterate	15	3.6
	Primary school (1 – 8)	16	3.9
	Secondary school (9 – 12)	130	31.5
	Above secondary school	252	61.0
Religion	Protestant	212	51.3
	Orthodox	162	39.2
	Muslim	38	9.2
	Other^*^	1	0.3
Average monthly income^*^	<2629ETB	184	44.6
	≥2629ETB	229	55.4
Types of employment	Permanent	201	48.7
	Temporary	212	51.3
Family size^**^	Less than 4	178	43.1
	Greater than or equal 4	235	56.9
Types of work	Office	91	22.0
	Onsite	322	78.0
Position at work	Yes	13	3.1
	No	400	96.9

Other religion: Adventist ^**^based on Ethiopia Demographic and Health Survey (EDHS) ^*^based onWorld Bank poverty line cut point

### Organizational related characteristics of respondents

Different organizational related variables were assessed. From the total respondents half of the participants 210 (50.8%) had reported poor organizational support. Most participants, 295 (71.4%) had poor working conditions and 286 (69.2%) of the study participants reported as they had poor organizational job security. More than half 225 (54.5%) of the participant reported that they got poor recognition on their jobs from the organization (Table 2).

**Table 2:** Organizational related characteristics of employees in Hawassa industrial park in Hawassa city, South Ethiopia, September, 2021 (*N* = 413)

Variables	Categories	Frequency(n)	Percentage (%)
Organizational support	Good	203	49.2
	Poor	210	50.8
Working condition	Good	118	28.6
	Poor	295	71.4
Organizational job security	Good	127	30.8
	Poor	286	69.2
Employees recognition	Good	188	45.5
	Poor	225	54.5
Work experience in years^a^	< 2 ^1^/_2_ years	148	35.8
	> 2 ^1^/_2_ years	265	64.2
Working hour per week^b^	< 48 hr	394	95.4
	> 48 hr	19	4.6
Overtime working hr. per month^b^	< 20 hr	76	18.4
	> 20 hr	337	81.6

^a^categorized by mean

^b^Based on Ethiopian labour proclamation 377/2003

### Job content and clinical characteristics of respondents

Regarding the job-related problems more than one-third 187 (45.3%) of respondents had high time pressure on their job and more than half 214 (51.8%) of participants reported low job control at their workplace. More than three fourth 313 (75.8%) of study participants reported poor learning opportunities and more than half 238 (57.6%) reported poor physical condition of a working environment. The study reveals almost half 196 (47.5%) of respondents reported the presence of workplace violence. The study also reveals 33 (8.0%) of participants reported as they have chronic medical illness: - hypertension [8] (1.9%), diabetes [2] (0.5%), HIV/AIDS [8] (1.9%) and kidney disease [15] (3.6%) (Table 3).

**Table 3:** Job content and clinical characteristics of employees in Hawassa industrial park in Hawassa city, South Ethiopia, September, 2021 (*N* = 413)

Variables	Categories	Frequency(n)	Percentage (%)
Time pressure	Low	226	54.7
	High	187	45.3
Attention demand	Low	232	56.2
	High	181	43.8
Job control	High	199	48.2
	Low	214	51.8
Resource in working team	Enough	234	56.7
	Scarcity	179	43.3
Learning opportunities	Good	100	24.2
	Poor	313	75.8
Interaction with machine	Good	94	22.8
	Poor	319	77.2
Physical condition	Good	175	42.4
	Poor	238	57.6
Work place violence	No	217	52.5
	Yes	196	47.5
Chronic medical illness	No	380	92.0
	Yes^a^	33	8.0
Current illness	Yes	292	70.7
	No	121	29.3

^a^presence of hypertension, diabetes, HIV/AIDS or kidney disease

### Substance use characteristics of respondents

**Fig. 1:**
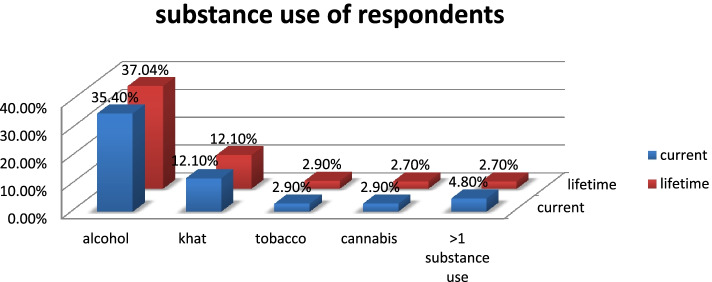
Substance use characterstics of of employees in Hawassa industrial park in Hawassa city, South Ethiopia, September, 2021 (*N* = 413)

From the total study participants more than half 227 (54.9%) had a history of any substance use at least once in their lifetime, while 153 (37.04%) of them uses alcohol and 50 (12.1%) of them use Khat. Almost half 219 (53.02%) of respondents had a history of any substance use in the past 3 months, of which 146 (35.4%) use alcohol and 50 (12.1%) uses Khat (Fig. 1).

### Prevalence of work-related stress among employees of industrial park

The overall prevalence of work-related stress in this study was 196 (47.5%) with 95% CI (43.2, 52.1). Among those who have work-related stress 53 (27.04%) were males and 143(72.96%) were females.

### Factors associated with work related stress among employees of industrial park

Bivariate and multivariable analysis was done to see factors associated with work-related stress. Hence, types of employment, family size, organizational support, working condition, work experience, learning opportunity, physical environment, workplace violence, current use of alcohol, current use of khat, and current use of more than one substance were found to be associated with work-related stress on bivariate analysis and entered to multivariate analysis. Variables associated with work-related stress on bivariate analysis were checked for multicollinearity before the final model, and all the candidates for final models had Variance Inflation Factor (VIF) less than 1.2 and tolerance of greater than 0.87. Therefore, there was no problem with collinearity. Multivariable logistic regression analyses have revealed that temporary employment, poor working condition, work experience less than two and half years, poor learning opportunity, poor organizational support, current use of khat, and current use of alcohol were significantly associated with work-related stress.

The finding from this study shows that temporary employees had a 60% reduced risk of work-related stress AOR = 0.40, 95% CI (0.26–0.63) than permanent employees. The study also reveals having poor working conditions was about 2.1 times more likely to have work-related stress AOR = 2.10, 95% CI (1.30–3.39) than having good working conditions. Additionally, employees who have work experience less than two and half year were about 3.22 times more likely to have work-related stress than employees who have work experience greater than two and half years AOR = 3.22, 95% CI (2.01–5.12). Similarly, employees who have poor organizational support were about 1.59 times more likely to have work-related stress AOR = 1.59, 95% CI (1.02–2.48) than those employees who have good organizational support.

In addition, the odds of having work-related stress was 1.94 times higher AOR = 1.94, 95% CI (1.12–3.22) among employees who had poor learning opportunities as compared with employees who had good learning opportunities. Employees who report the current use of khat was about 2.35 fold more likely to have work-related stress AOR = 2.35, 95% CI (1.19–4.67) than nonusers. This study has also revealed that employees who have current use of alcohol were around times more likely to have work-related stress AOR = 2.14, 95% CI (1.35–3.34) than employees who didn’t report current use of alcohol (Table 4).

**Table 4:** Bivariate and multivariable analysis of factors associated with work-related stress among employees of Hawassa industrial park in Hawassa city, South Ethiopia, September, 2021 (*N* = 413)

Variables	Categories	Work related stress		COR & 95%CI	AOR & 95%CI	*P*-value
		Yes (%)	No (%)			
Types of employment	Permanent	107(53.2)	94(46.8)	1	1	1
	Temporary	89(42.0)	123(58.0)	1.57(1.07–2.32)	0.40(0.26–0.63)	< 0.001^*^
Family size	< 4	92 (51.7)	86 (48.3)	1	1	1
	> 4	104(43.3)	131 (55.7)	0.74 (0.50–1.10)	0.77(0.48–1.24)	0.285
Organizational support	Good	83(40.9)	120(59.1)		1	1
	poor	113(53.8)	97(46.2)	1.68(1.14–2.49)	1.59(1.02–2.48)	0.039^*^
Work experience in years	< 2^1^/_2_ year	88(59.5)	60(40.5)	2.13(1.42–3.21)	3.22(2.01–5.12)	< 0.001^*^
	> 2^1^/_2_ year	108(40.8)	157(59.2)	1	1	1
Learning opportunity	Good	44(38.9)	69(61.1)	1	1	1
	Poor	152(50.7)	148(49.3)	1.61 (1.04–2.50)	1.94(1.12–3.22)	0.011^*^
Working condition	Good	41(34.7)	77(65.3)	1	1	1
	Poor	155(52.5)	140(47.5)	2.07(1.33–3.23)	2.10(1.30–3.39)	0.003^*^
Physical environment	good	71(40.6)	104(59.4)	1	1	1
	poor	125(52.5)	113(47.5)	1.62(1.09–2.40)	1.51(.97–2.36)	0.070
Workplace violence	No	90(41.5)	127(58.5)	1	1	1
	Yes	106(54.1)	90(45.9)	1.66(1.13–2.45)	1.38(0.87–2.20)	0.173
Current use of khat	No	165(45.5)	198(54.5)	1	1	1
	Yes	31(62.0)	19(38.0)	1.96(1.07–3.59)	2.35(1.19–4.67)	0.014^*^
Current use of alcohol	No	111(41.6)	156(58.4)	1	1	1
	Yes	85(58.2)	61(41.8)	1.96(1.30–2.95)	2.14(1.35–3.34)	0.001^*^
Current use of > 1 substance	No	183(46.6)	210(53.4)	1	1	1
	Yes	13(65.0)	7(35.0)	2.13(0.83–5.46)	1.05(.34–3.24)	0.934

^*^Variables with significant association at *p*-value < 0.05, 1 = reference category

## Discussion

Stress in the workplace is a worldwide public health problem. Studies in African countries focusing on work stress, especially among industrial park workers, are scarce. The overall prevalence of work-related stress among employees was found to be 47.5% with 95% CI (43.2, 52.1). This finding was comparable with those studies done among vehicle repair workers in India [28] and Bahirdar textile factory workers, Ethiopia [15] where the preavalence of work-related stress was reported as 47 and 45.2% respectively.

Even so, the finding of the current study was higher than those studies done in Dukem shoe manufacturing, Ethiopia, 40.4% [16], democratic republic of Congo, 28% [14], India, 25% [29], Iran, 21.3% [13], Thailand, 27.5% [10], Bristol City, 20% [30] and Vietnam, 20.7% [31]. The reported discrepancies might be explained by that developed countries have organized safety precautions and facilitated access in advance to health and safety training with a better socio-economic status. They have also better-improved levels of health-care services and enforcement regulations than developing countries (except for those Dukem shoe manufacturing and congo) [32]. For the study done in Congo variation in sociocultural, study setting, and tools used to assess work-related stress might be possible explanations. They have used self-administered (Karasek and Siegrist’s scale) while interviewer-administered (workplace stress scale) was used for the current study. In addition, the discrepancy observed with the study done at Dukem shoe manufacturing might be possibly explained by difference in study setting.

However, the current study finding is lower than studies done in Pakistan among medical educators [33] and Iran among nurses [34] where the prevalence of work-related stress was reported as 94, and 68% respectively. The possible explanation for the difference observed might be the variation of the study population and the difference in sample size. The first study was done among 111 medical educators in one private college. Most of the educators in this college take on their teaching role in addition to their clinical activities, which might lead the individuals to more stressful conditions. In the study done among nurses in Iran, the sample size was 250 and they used different tools (OSIPOW) which assess three dimensions of work adjustment, occupational stresses, psychological strain, and coping resources to assess stress at the workplace. In addition, the work nature of their study population had high workloads and needs the delivery of empathetic, culturally sensitive, proficient, and moral care in the working environment with increasing responsibility. On that account, the stated factors might be the possible explanation for the discrepancy observed with the current study.

In the present study, temporary employment was found to be negatively associated with work-related stress among employees. This finding is reconcilable with studies done in Norway [35] and Japan [36]. This detection might be explained by that the average level of effort and effort-reward imbalance which may have been influenced by their work is higher among permanent workers than temporary. In addition, permanent workers might be influenced by a commitment that the employer has made to them by entering into a permanent employment agreements, which might expose them to more pressure to achieve [37].

Outcomes from this study reveal that poor organizational support for employees was found to be an independent predictor of work-related stress. This is in line with the studies done in Nigeria [38], Sweden [39], and Dukem, Ethiopia [16]. The possible explanation for this result might be employees whose employers provide insufficient support are often frustrated, apathetic, and might have poor achievement. As a consequence, this may lead to unsafe work practices, increased staff turnover, and even illness [7].

In the current study having work experiences less than two and half years was found to be significantly associated with work-related stress among employees. This is congruous with studies done in Egypt [40] and Dukem, Ethiopia [16]. Less experience of interaction with a machine, working environment, and getting new experience might be the possible explanation for the association found.

This study also reveals poor learning opportunities as independent predictors of work-related stress among employees. The findings from the study conducted in Malaysia [41] and Gondar, Ethiopia [42] concur with the association. Dealing with a certain fear of not having career development due to a lack of upgrading educational status resulting in absence of learning opportunities might be the possible explanation for the finding [43].

Finding from this study indicates poor working condition was a risk factor for having work-related stress among employees. This result is agreeing with the study done in Iran [44], India [45], and Ghana [46]. The possible explanation for this association might be that employees who have a discordant relation ship with supervisors, colleagues and also uncomfortable feelings towards their work will have more stress related to their job than others.

In the current study, current use of khat had a significant positive association with work-related stress among employees. The result observed in studies conducted at the bahirdar textile factory [15] and the amhara region, Ethiopia [47] is consistent with the finding. The time spent chewing khat may influence the working time of the individuals and also as the employees spent more time chewing khat they might experience the feeling of guilty and self-blame, which leads to frustration and stress in the long run [47].

In the present study current use of alcohol was also found to be independent predictor of work-related stress. This finding is consistent with studies conducted in the USA [48] and Germany [49]. The Psychosocial and direct effects of alcohol on the brain might result in stress among employees. In addition, inconsistencies of employees’ work performance and rewards related to that can cause unanticipated negative consequences among employees and this may lead to starting alcohol use as a coping [50].

## Conclusion

The prevalence of work-related stress among employees of Hawassa industrial park was high. Temporary employment, poor working condition, work experience less than two and half years, poor learning opportunity, poor organizational support, current use khat and current use of alcohol were significantly associated with work-related stress. Enhancing stress management skills and primary prevention on identified risk factors was recommended.

## Data Availability

The datasets used and/or analyzed during the current study are not publicly available to keep confidentiality of study participants but are available upon reasonable request from the corresponding author.

## Abbreviations

AOR: Adjusted Odds Rratio
CI: Confidence interval
COR: Crude Odds Ratio
SD: Standard deviation
SE: Standard error
SPSS: Statistical package for social sciences
WPSS: Workplace Stress Scale
WRS: Work-related Stress
